# Does living alone exacerbate depression in older adults?

**DOI:** 10.3389/fpsyg.2025.1553080

**Published:** 2025-02-25

**Authors:** Haolin Wang, Bing Sun

**Affiliations:** School of Finance and Public Administration, Anhui University of Finance and Economics, Bengbu, China

**Keywords:** living arrangements, older adults, depression, family cultures, intergenerational gap

## Abstract

**Introduction:**

Living alone, which has become increasingly common in China, weakens the emotional connections between parents and children—fundamental family functions in Confucianism. This trend has raised concerns regarding depression among older adults living alone. Numerous researchers have evaluated the influence of living alone on depression among older adults in different cultures. However, the consensus has yet to be reached.

**Methods:**

This study adopted a fixed effects model to analyze three sets of data from the China Health and Retirement Longitudinal Study based on different family cultures reflected by living arrangements.

**Results:**

Living alone reduced depression among older adults by 0.267 (CES-D, 10–40). The effects of living alone on depression among older adults predominantly originate from living close to their children. This lifestyle effectively balances the need for individual independence and the demand for maintaining tight family relationships and reconciles intergenerational family conflict. Differences between rural and urban areas were also identified. This search indicated that living alone improved depression in rural older adults; however, no significant effects were found for urban older adults.

**Discussion:**

As living close to their children is conducive to improving depression among older adults, policymakers are advised to focus on local employment. Special emphasis should be placed on incentivizing migrant workers to return to their rural hometowns for entrepreneurship or employment.

## 1 Introduction

In East Asian cultures, it is traditional for older adults to live with their children, a practice regarded as a symbol of filial piety in Confucianism (Croll, 2006). However, this tradition has been challenging in China. According to modernization theory, as societies become more modernized, there will be a corresponding decrease in household size and an increase in older adults living separately from their children (El-Ghannam, 2001). According to China’s 1982–2020 population census, household size shrank, and intergenerational family structure became simpler. Even the universal implementation of the two-child policy in China has failed to change these trends. In 1982, the average household size was 4.41 persons, which decreased to 2.62 in 2020. In 1982, families with only one generation accounted for 13.9% of all families; this percentage increased to 49.5% in 2020. In 1982, although families with two generations accounted for 48.2% of all families, this percentage declined to 36.7% in 2020 (Ma, 2023). Indeed, in China, it is increasingly common for older adults to live only with their spouses or alone. Living alone has become a major living arrangement for older adults in China.

From a global perspective, older adults’ living arrangements have been identified as a significant component of healthy aging (Reher and Requena, 2018). The growing popularity of living alone among older adults in China has somewhat jeopardized the traditional “intergenerational feedback model”^1^ in Confucianism (Zhou, 2021). This weakens the emotional connections between parents and children, which is hindered by distance, and has raised concerns about the depression of older adults who live alone. Older adults are more vulnerable than young people. They are more susceptible to mental illnesses caused by external factors (Wild et al., 2014). As China is undergoing compressed modernization and a compressed aging process, the conflicts between tradition and modernity are partially reflected in older adults’ living arrangements. Traditional culture, which requires filial piety and intergenerational co-residence, coexists with modern culture, which values freedom and independence. Consequently, many researchers have associated living arrangements, reflecting Chinese social changes, with depression in the older adult population. Nevertheless, this conclusion remains controversial (Gu et al., 2018; Xu et al., 2019).

The fundamental cause of this disagreement lies in the complicacy of the relationship between living arrangements and depression, as living with their children has both benefits and disadvantages for older adults (Sakurai et al., 2019). Family support theories maintain that living with adult children helps older adults obtain economic, emotional, and other support, which fosters harmonious family relationships (Chen and Short, 2008). Living arrangements are vital environmental elements and resources that enable older adults to make requests from their cohabitants, significantly influencing their lifestyles, cognitive functions, and mental health. In the long-maintained tradition in China that prioritizes filial piety, living with adult children is considered the ideal living arrangement for older adults (Sereny and Gu, 2011). Xu et al. (2022) utilized the Chinese Longitudinal Healthy Longevity Study (CHARLS) for research and found that older adults living alone were 1.26 times more likely to experience depression than those who lived with family members. Similar findings were observed in studies of Koreans (Kim and Choi, 2011; Do and Malhotra, 2012), Vietnamese (Ken and Teerawichitchainan, 2015), and Japanese (Tiedt, 2013). Familyism, which is upheld by East Asian cultures (Luna et al., 1996), strongly influences the living arrangements of older adults.

Family conflict theory claims that parents encounter challenges when co-residing with their children. Differences in lifestyles and notions between generations may cause conflicts, weakening the benefits of intergenerational support and impairing older adults’ welfare (Rook, 1984). One reason for family conflicts arising from intergenerational co-residence is the generational gap. China’s opening has brought about dramatic societal changes, leading to distinct social backgrounds between generations. Accordingly, the generational gap has become a vital feature of intergenerational relationships. There are differences in notions and behavioral patterns between generations (Zhou, 1994). Grandchildren’s education easily triggers family conflict when older adults live with their children in China (Goh and Kuczynski, 2010; Goh and Kuczynski, 2012).

Moreover, multi-generational co-residence complicates family relationships, which may induce competition regarding family status, authority, and even intimate relationships between generations. For example, the relationship between a mother-in-law and her daughter-in-law is a major trigger for family conflict in China (Shen, 2023). Research shows that family conflict is detrimental to the mental health of older adults who live with their children (Huang et al., 2020). Ren and Treiman (2015) examined the data from the 2010 China Family Panel Studies (CFPS) and found that older adults who lived with their adult children were less happy and more depressed than those who lived with spouses.

The two theories explain the positive and negative effects of co-residence on depression among older adults. According to the family-support theory, co-residence is convenient for family members to provide emotional support to older adults. Confucianism regards multigenerational co-residence as a sign of happiness, and co-residence can play a role in suggesting happiness to older adults. Confucianism calls for a strict pecking order based on age in the family, which gives older adults authority and restrains the impact of intergenerational conflicts on depression among older adults. According to family conflict theory, because of differences in living habits and notions, co-residence may cause intergenerational conflicts. The implicit assumption is that the strict pecking order begins to fail. The two theories take co-residence as the object of discussion and explain its influence on depression among older adults through different cultural concepts, which position living alone in direct contrast to co-residence. Intergenerational relationships in Chinese families present a “duality” where traditional cultural resilience and the tensions caused by divergent intergenerational notions coexist. Intergenerational responsibility and ethics carry a binding force over individual behavior in family life, laying the basis for the traditional “intergenerational feedback model.” However, as older adults and their adult children hold different notions, these divergences cause intergenerational family conflicts (Xiao and Guan, 2018). Those theories ignore individuals’ adaptive choices when confronted with “duality.” The popularity of the “distance from a bowl of porridge” living arrangement reflects their choice, which may dispel the anxiety caused by “duality.”

Our study makes significant contributions to the literature. There is bidirectional causality between living arrangements and depression (Gu et al., 2018). This induces endogeneity, which has not been well addressed in the previous literature. The marginal contribution of this study lies in addressing endogeneity using instrumental variables and panel data. This study breaks through the dominant “dichotomy” classification, where living arrangements are classified into “living with their children” and “living alone.” “living alone” is further classified into “living close”^2^ and “living far”^3^. Living close effectively balances the need for personal independence with the need to maintain close relationships, which reflects the strategic and adaptive choices made by families in the face of the “duality” of intergenerational relationships. The new classification accurately portrays older adults’ living arrangements, which is conducive to further analyzing the relationship between living arrangements and depression in China.

## 2 Data and methods

### 2.1 Data

The data used in this study were obtained from CHARLS, which aims to collect a set of high-quality microdata on people aged 45 years and above and their families to analyze aging in China and facilitate cross-discipline research on the subject. The national baseline survey of CHARLS was initiated in 2011 and covered 17,000 individuals in approximately 10,000 households across 450 villages in 150 counties. Through detailed queries regarding older adults’ living arrangements and health conditions, we collected rich demographic information and derived satisfactory data for the analysis in this research. We used longitudinal data from three consecutive waves (2013, 2015, 2018) of the CHARLS survey, including 58,985 samples. Our analysis specifically targeted individuals aged 60 years and above, with at least one living child, undergoing continuous investigation on three occasions. The dataset comprised 14,739 samples after removing samples with missing variables.

### 2.2 Variables

The Center for Epidemiologic Studies Depression Scale (CES-D) was developed by the United States National Institute of Mental Health in 1977. It has been widely applied in epidemiological surveys. The CHARLS applied its simplified version to measure depression among older adults. The scale comprises 10 items related to depressive emotions and behaviors that respondents feel or engage in during the week prior to the time when they take the test. Among the items, the fifth item, “I was hopeful about the future,” and the eighth item, “I was happy,” expressed positive emotions, whereas the other eight items conveyed negative feelings. Therefore, the scores for the two items were separately reversed. Subsequently, we aggregated the scores for all 10 items to indicate older adults’ depression levels. The total CES-D score ranges between 10 and 40, with a higher score indicating a higher level of depression. A total score of 20 and above indicates depression in the respondent (Björgvinsson et al., 2013).

Regarding older adults’ living arrangements (independent variable), we divided them into “living with children” and “living alone” and regarded “living with children” as the control group. We categorized people whose children answered “living with parents and having financial dependence” and “living together but having financial independence” in the CHARLS questionnaire into the “living with children” group, while the rest were categorized into the “living alone” group.

We selected four types of control variables. The first category comprises various demographic characteristics vis-à-vis older adults, including gender, age, marital status, household registration, years of education, work, and number of children. The second category comprises older adults’ social and economic features, including cross-generational childcare, average family income, financial support from children, and their medical insurance and pensions. The third category comprised older adults’ health conditions, including drinking, smoking, chronic diseases, and overall health status. In this research, “drinking” is defined as consuming alcoholic beverages on more than one occasion per month. Accordingly, a value of 1 was assigned to this variable if the criterion was met and 0 otherwise. The fourth category comprises respondents’ social engagement. A total of 11 activities were listed in the CHARLS questionnaire: Have you participated in any of the following social activities over the past month? In this research, a value of 1 was assigned to this variable if a respondent had participated in one social activity and 0 otherwise.

### 2.3 Analysis strategies

We utilized longitudinal data, which requires a Hausmann test to determine whether to select a random effects model or a fixed effects model. The *p* value of the test was 0.0000, which rejected the null hypothesis, so the fixed effect model was chosen. The equation was as follows:

(1) $$Y_{\mathrm{it}}=\beta_{1}\mathrm{livin}g_{\mathrm{it}}+\beta_{2}X_{\mathrm{it}}+a_{i}+\in_{\mathrm{it}}$$

In Equation 1, $Y_{\mathrm{it}}$ , $\mathrm{livin}g_{\mathrm{it}}$ , and $X_{\mathrm{it}}$ represent the depression level, living arrangements, and control variables, respectively, during period *t*; $a_{i}$ denotes individual-specific intercepts, and $\in_{\mathrm{it}}$ represents random errors.

The four categories of effect factors were sequentially introduced into Models 1–4, and panel regression analysis was conducted. All four models simultaneously controlled for individual FE(Fixed Effect) and time FE to examine the association between older adults’ living arrangements and their depression, which helped control for observed or unobserved individual features that did not change over time and temporal factors independent of individual changes, such as regional culture.

In empirical research on the influence of living arrangements on depression, addressing the potential endogeneity caused by reverse causality is essential. Older adults suffering from depression tend to live with their children to receive care (Gu et al., 2018). Therefore, reverse causality may exist between living arrangements and depression. The current quantitative research did not consider the endogeneity caused by reverse causality, so we used an IV(Independent Variable) to conduct the robustness test.

## 3 Results

### 3.1 Descriptive statistics

The control variables in this study are divided into time-varying and time-constant covariables. Time-constant covariables are not shown in the regression results but are still in descriptive statistics. Tables 1, 2 show that the average depression level in the samples was 18.819 (< 20), indicating a generally good depression status. Of the older adults, 60.1% lived alone, while only 39.9% lived with their children. The depression level of older adults who lived alone was lower than average, reaching 18.62, while that of older adults who lived with their children was higher than average, at 19.13.

**Table 1:** Descriptive statistics.

Variable	Description	Mean	S.D.	Min	Max
Dependent variable					
Depression	Depression level	18.819	6.558	10	40
Independent variable					
Living arrangements	Living with children = 0; Living alone = 1	0.601	0.490	0	1
Time-constant covariables					
Gender	Female = 0; Male = 1	0.500	0.500	0	1
Years of education	Illiterate or not graduating from primary school = 0, primary school education = 6, junior high school education = 9, senior high school education or technical secondary education = 12, college degree or above = 15	4.981	3.866	0	15
Time-varying covariables					
Age	Age	69.085	6.112	60	108
Marital status	No spouse = 0; Having a spouse = 1	0.812	0.391	0	1
Household registration	Rural = 0; Urban = 1	0.214	0.410	0	1
Work	Have you worked in the past year	0.553	0.497	0	1
Number of children	Number of children who are alive	3.402	1.530	1	15
Cross-generational childcare	Weeks of intergenerational care provided for grandchildren in the last year	14.335	22.044	0	52
Average personal income of the family	Average personal income of the family (yuan per year)	5075.519	18255.365	0	1,007,500
Financial support from children	Financial support received from children in the past year (yuan per year)	2517.074	9345.481	0	515,000
Medical insurance	Non-medical insurance holder = 0; Medical insurance holder = 1	0.978	0.147	0	1
Pension	Non-pensioner = 0; Pensioner = 1	0.863	0.344	0	1
Drinking	Non-drinker = 0; drinker = 1	0.253	0.435	0	1
Smoking	Non-smoker = 0; smoker = 1	0.461	0.499	0	1
Chronic diseases	No chronic diseases = 0; With a chronic disease = 1	0.740	0.439	0	1
Overall health status	Wonderful = 1; Quite good = 2; Good = 3; Average = 4; Bad = 5; Awful = 6	3.736	1.035	1	6
Social engagement	No social activity = 0; Having participated in social activities = 1	0.499	0.500	0	1

**Table 2:** Descriptive statistics of samples based on living arrangements.

Variable	Living with children (Mean)	Living alone (Mean)	*T* test
Dependent variable			
Depression	19.130	18.620	0.508***
Time-constant covariables			
gender	0.492	0.506	−0.014*
Years of education	4.793	5.105	−0.313***
Time-varying covariables			
Age	68.510	69.460	−0.950***
Marital status	0.772	0.839	−0.066***
Household registration	0.186	0.232	−0.046***
Work	0.563	0.546	0.017**
Number of children	3.463	3.361	0.102***
Cross-generational childcare	18.55	11.53	7.023***
Average personal income of the family (logarithm)	5.357	5.279	0.078
Financial support from children (logarithm)	5.530	6.291	−0.761***
Medical insurance	0.976	0.979	−0.004
Pension	0.854	0.868	−0.014**
Drinking	0.233	0.266	−0.033***
Smoking	0.455	0.466	−0.011
Chronic diseases	0.747	0.736	0.011
Overall health status	3.791	3.700	0.090***
Social engagement	0.490	0.506	−0.016**

In all tables, asterisks indicate statistical significance. *** Is significant at the 1% level, ** at the 5% level, and * at the 10% level.

Regarding demographic features, the average age of older adults was 69.085 years, with males, individuals with spouses, and rural residents accounting for 50, 81.2, and 21.4%, respectively. On average, older adults only received 4.981 years of education, 55.3 percent of older persons had worked in the past year, the average number of children was as high as 3.402, and the average number of cross-generational childcare provided for grandchildren was 14.335 weeks. Regarding social and economic features, the average annual income of individuals in the families was 5075.529 yuan, and financial support received from children in the past year averaged 2,517.074 yuan. Of the older adults, 97.8% had medical insurance, and 86.3% were granted pensions. In terms of physical health, 25.3% were drinkers, 46.1% were smokers, and 74.0% had chronic diseases. The overall health status was between “good” and “average” (3.736). Regarding social engagement, 49.9% of the respondents said they participated in at least one social activity. Generally, descriptive statistics supported the hypothesis that older adults’ living arrangements impacted their depression.

### 3.2 Regression analysis

The coefficient in Model 1 is presented in the second row of Table 3, which estimates the relationship between older adults’ living arrangements and depression when their demographic characteristics are controlled for. According to Model 1, living alone is more beneficial for improving depression among older adults than living with their children, as older adults who lived alone scored 0.287 lower than those who lived with their children. Older adults with spouses were healthier than those without spouses (*β* = −1.079). Urban older adults experienced a lower depression level than rural older adults (*β* = −0.694). Meanwhile, older adults who had worked in the past year were in better health than those who had not (*β* = −0.503). Compared with Model 1 in Table 3, the results of Model 2 are still robust after controlling for social and economic factors. The results of Model 3 were still robust after controlling for health condition factors. Compared to older adults who do not drink, older adult drinkers show a lower depression level (*β* = −0.561), and drinking benefits them. Model 4 remained robust after controlling for the social engagement factor. Participating in social activities negatively affects the depression level (*β* = −0.298), which improves depression among older adults. Therefore, living alone is negatively correlated with depression levels, and these associations are statistically significant at the 5% level, which suggests that living alone is more conducive to improving depression among older adults.

**Table 3:** FE analysis of the influence of living alone on depression among older adults.

Variable	(1)	(2)	(3)	(4)
	Model 1	Model 2	Model 3	Model 4
Living alone	−0.287^**^	−0.292^**^	−0.272^**^	−0.267^**^
	(−2.35)	(−2.31)	(−2.17)	(−2.13)
Age	0.004	−0.000	−0.006	−0.008
	(0.09)	(−0.01)	(−0.14)	(−0.20)
Marital status	−1.079^***^	−1.219^***^	−1.148^***^	−1.174^***^
	(−3.87)	(−4.21)	(−3.99)	(−4.08)
Household registration	−0.694^*^	−0.752^*^	−0.845^**^	−0.839^**^
	(−1.78)	(−1.89)	(−2.14)	(−2.13)
Work	−0.503^***^	−0.555^***^	−0.459^***^	−0.455^***^
	(−3.69)	(−3.92)	(−3.26)	(−3.23)
Number of children	0.124	0.112	0.122	0.120
	(1.41)	(1.21)	(1.33)	(1.30)
Cross-generational childcare		−0.002	−0.001	−0.001
		(−0.57)	(−0.40)	(−0.37)
Average personal income of the family (logarithm)		0.013	0.015	0.016
		(0.85)	(0.95)	(1.03)
Financial support from children (logarithm)		−0.010	−0.006	−0.003
		(−0.50)	(−0.29)	(−0.16)
Medical insurance		−0.297	−0.234	−0.229
		(−0.87)	(−0.69)	(−0.68)
Pension		−0.095	−0.076	−0.073
		(−0.66)	(−0.53)	(−0.51)
Drinking			−0.561^***^	−0.546^***^
			(−3.21)	(−3.13)
Smoking			0.024	0.012
			(0.07)	(0.03)
Chronic diseases			0.156	0.157
			(1.27)	(1.27)
Overall health status			0.738^***^	0.734^***^
			(11.16)	(11.10)
Social engagement				−0.298^***^
				(−2.72)
_cons	19.453^***^	19.898^***^	17.031^***^	17.386^***^
	(6.92)	(7.00)	(5.99)	(6.11)
Whether individual FE is controlled for	Yes	Yes	Yes	Yes
Whether time FE is controlled for	Yes	Yes	Yes	Yes
*N*	14,739	14,739	14,739	14,739

### 3.3 Robust test: reverse causality and measurement of the independent variable

As older adults’ living arrangements may be influenced by their depression, the problem of the reverse causality should be tackled in the model. IV may be used to address this problem, thereby enhancing the validity and consistency of the estimation results. Referring to Cai et al. (2023), we selected the percentage of older adults living alone in the community as IV, which was associated with living arrangements while having no correlation with the depression among older adults. The Cragg-Donald Wald F statistic (2513.557) significantly exceeded the Stock-Yogo 10% critical value (16.38), which rejected the hypothesis of a “weak IV.” Table 4 presents the results of the final regression analysis. Compared to living with their children, living alone was more helpful in reducing depression levels among older adults (*β* = −1.398). This indicates that the results are robust.

**Table 4:** IV test.

Variable	Coefficient
Percentage of older adults living alone	−1.398^***^ (−5.19)
Control variables	Control
Whether individual FE is controlled for	Yes
Whether time FE is controlled for	Yes
*N*	14,739

Owing to rural labor migration, generation-skipping living arrangements are prevalent, where older adults may be pressured to care for their grandchildren, and they can get emotional support from close relationships with their grandchildren, which was ignored in the measurement of the independent variable. Information about generation-skipping living arrangements is not available directly from CHARLS. We can obtain information about living with children and caring for grandchildren from CHARLS. Not living with children but caring for grandchildren for more than 26 weeks (two quarters) is considered a generation-skipping living arrangement. The test results remained robust after deleting generation-skipping living arrangement samples. The results remain robust after revising the selection criteria to 39 weeks (three quarters) (Table 5).

**Table 5:** Analysis of the generation-skipping living arrangement.

Variable	26 weeks (two quarters)	39 weeks (three quarters)
Living alone	−0.333^**^	−0.322^**^
	(−2.18)	(−2.15)
Control variables	Control	Control
Whether individual FE is controlled for	Yes	Yes
Whether time FE is controlled for	Yes	Yes
*N*	12,717	12,828

### 3.4 Strategic choice of living arrangements

Living arrangements are categorized into two styles based on the binary opposing thinking that traditional family culture opposes modern culture. Families with older adults want to preserve strong bonds and support from traditional families. They also hope to enjoy freedom from family interference. To address the “duality” of intergenerational relationships, they strategically choose to live close to their children but in a separate home to accommodate and balance the contradiction. Consequently, to precisely portray the cultural phenomena revealed in living arrangements, we further categorized living alone into “living close” and “living far.” The results are shown in Table 6, which indicates that throughout Models 1–4, older adults living close by are significantly less depressed, at the 1% level, than those who live with their children. Living far away was also negatively related to the depression level of older adults, but was insignificant. Thus, the significant impact mainly stems from living close rather than distant. This strategic choice to balance the “duality” of intergenerational relationships proves to be a good solution.

**Table 6:** Analysis of three types of living arrangements.

Variable	Model 1	Model 2	Model 3	Model 4
Living close	−0.398^***^	−0.398^***^	−0.377^***^	−0.373^***^
	(−2.84)	(−2.76)	(−2.63)	(−2.60)
Living far	−0.155	−0.165	−0.146	−0.142
	(−1.05)	(−1.09)	(−0.97)	(−0.94)
Age	0.003	−0.000	−0.006	−0.008
	(0.09)	(−0.01)	(−0.14)	(−0.20)
Marital status	−1.087^***^	−1.226^***^	−1.155^***^	−1.181^***^
	(−3.90)	(−4.23)	(−4.02)	(−4.11)
Household registration	−0.697^*^	−0.756^*^	−0.848^**^	−0.843^**^
	(−1.79)	(−1.90)	(−2.15)	(−2.14)
Work	−0.502^***^	−0.554^***^	−0.458^***^	−0.454^***^
	(−3.68)	(−3.91)	(−3.25)	(−3.23)
Number of children	0.126	0.114	0.124	0.121
	(1.43)	(1.23)	(1.34)	(1.32)
Cross-generational childcare		−0.002	−0.001	−0.001
		(−0.58)	(−0.41)	(−0.38)
Average personal income of the family (logarithm)		0.014	0.015	0.016
		(0.88)	(0.98)	(1.06)
Financial support from children (logarithm)		−0.011	−0.007	−0.004
		(−0.55)	(−0.34)	(−0.22)
Medical insurance		−0.309	−0.247	−0.242
		(−0.91)	(−0.73)	(−0.71)
Pension		−0.095	−0.076	−0.073
		(−0.66)	(−0.53)	(−0.51)
Drinking			−0.566^***^	−0.551^***^
			(−3.24)	(−3.15)
Smoking			0.024	0.013
			(0.07)	(0.04)
Chronic diseases			0.153	0.153
			(1.24)	(1.24)
Overall health status			0.738^***^	0.734^***^
			(11.15)	(11.10)
Social engagement				−0.297^***^
				(−2.72)
_cons	19.448^***^	19.914^***^	17.053^***^	17.407^***^
	(6.92)	(7.00)	(6.00)	(6.12)
Whether individual FE is controlled for	Yes	Yes	Yes	Yes
Whether time FE is controlled for	Yes	Yes	Yes	Yes
*N*	14,739	14,739	14,739	14,739

### 3.5 Urban–rural differences

Family culture is key to older adults’ living arrangements. Disparities in family culture between urban and rural areas in China also influence living arrangements. Meanwhile, the urban–rural gaps in public cultural services, public healthcare services, and economic development also contribute to differences in depression among older adults. Therefore, to analyze the influence of living arrangements on depression among older adults, we categorized the original sample into rural and urban subsamples and conducted further analysis of differences. As shown in Table 7, the percentage of older adults living with their children in urban areas was 6.2% lower than that of older adults in rural areas, and the percentage of older adults living alone in urban areas was 6.2% higher than that of older adults in rural areas. The analysis of the three types of living arrangements indicated that the percentage of urban older adults living close by was 3.63% lower than that of their rural peers, and the percentage of urban older adults living far away was 9.84% higher than that of their rural peers. Both rural and urban older adults living alone showed lower depression levels than those who lived with their children. The analysis of the three types of living arrangements also revealed the same trends among urban and rural older adults, who suffered from a lower level of depression when they lived close to their children and a higher level of depression when they lived with their children.

**Table 7:** Description of urban–rural differences.

Variable	Rural	Urban	Rural	Urban	
	Proportion	Proportion	Depression level	Depression level	*T* test
Living with children	41.00%	34.79%	19.60	17.10	2.501^***^
Living alone	59.00%	65.21%	19.16	16.87	2.313^***^
Living with children	41.00%	34.79%	19.60	17.10	2.498^***^
Living close	28.79%	25.16%	18.95	16.79	2.205^***^
Living far	30.21%	40.05%	19.36	16.91	2.452^***^
Subsample	100%	100%	19.34	16.94	2.406^***^

This study analyzed the rural–urban differences in the effect of living arrangements. As shown in Table 8, the results indicate that living alone is conducive to depression in rural older adults, which was significant; living alone was not conducive to depression in urban older adults, which was insignificant.

**Table 8:** Analysis of urban–rural differences.

Variable	Rural	Urban	Rural	Urban
Living alone	−0.354^**^	0.123		
	(−2.44)	(0.48)		
Living close			−0.389^**^	−0.267
			(−2.34)	(−0.93)
Living far			−0.310^*^	0.539^*^
			(−1.76)	(1.85)
Control variables	Control	Control	Control	Control
Whether individual FE is controlled for	Yes	Yes	Yes	Yes
Whether time FE is controlled for	Yes	Yes	Yes	Yes
*N*	11,364	3,375	11,364	3,375

Compared to living with their children, living close to them significantly improved depression in rural older adults. However, the effect of living far away was insignificant. Living close also improved depression among urban older adults, which was insignificant; living far away exacerbated depression among urban older adults, which was insignificant. The opposite effects explained why living alone did not significantly influence depression among urban older adults.

## 4 Discussion

Although the impact of living arrangements on depression has been widely discussed, a consensus remains to be reached. These disparities may be attributed to different social environments, cultures and research approaches. Living arrangements reflect certain cultural phenomena. The same living arrangement may have opposite influences on depression among older adults under different cultural traditions. This study introduced family sociology and used representative national data and an FE model to examine the effects of living alone on depression among older adults based on the background of changing family cultures.

The results indicated that older adults living alone were less depressed than those living with their children. These findings contradict some conclusions (Xu et al., 2022; Meng et al., 2024), which indicated that older adults living alone were more prone to depression. This inconsistency could be attributed to the use of different databases, examining older adults aged 65 years and above, and analyzing cross-sectional data, which could not control the effects of unobserved or time-constant variables. Meanwhile, the important conclusions in this study are consistent with some research (Ren and Treiman, 2015). With China’s rapid economic and social development, older adults’ living arrangements and family cultures have fundamentally changed. However, living alone has not undermined the foundations of intergenerational support systems, as predicted by family-support theory, and there are still strong relationships between older adults and their non-cohabiting children. Older adults living alone have regular contact with non-cohabiting children and receive financial and emotional support. They maintain strong intergenerational ties. Traditional family culture adapts to new living arrangements, which reduces its negative impact on older adults (Jothikaran et al., 2020). Moreover, living alone may avoid the family conflict, which is believed to be the cause of negative effects of living with children according to Huang et al. (2020). The provision of care for grandchildren by older adults may lead to intergenerational conflict, which may worsen their depression. This care brings responsibility pressure to older adults living with their children, while older adults living alone, who occasionally care for their grandchildren, experience more family happiness. Older adults living alone are freed from care and family affairs and have more opportunities for social participation, which is a way to counter depression (Simone et al., 2015). “Square dancing” in China is popular, reflecting the great enthusiasm of older adults to participate in social activities.

The paper further divided “living alone” into “living close” and “living far” to examine strategic choices. The results showed that the strategic arrangement of living close was the most beneficial in improving depression among older adults, which aligned with the “duality” theory. Older adults who lived close to their children could enjoy personal freedom while being well cared for. The study also considered the potential heterogeneity of the results caused by urban–rural cultural differences. The test indicated that living alone was beneficial in improving depression among older adults, while the influence on depression among urban older adults was insignificant. The analysis of three types of living arrangements revealed that the urban–rural difference was mainly caused by living far worse than the depression among urban older adults. Living alone, especially living close, has a more significant positive effect on depression among older adults in the countryside than in urban areas. One possible explanation is that rural older adults have a strong sense of independence and prefer to live alone. However, the effect of living alone is insignificant for older adults in cities, which are characterized by a greater degree of openness and freedom. Therefore, in conservative rural areas, this explanation is not convincing. Another possible explanation, according to family conflict theory, is that rural family conflict is particularly intense in the context of cultural change. However, conflicts among urban families are less intense because of effective intergenerational communication and tolerance. Therefore, living arrangements have a significant effect on depression in rural older adults, and the effect on depression in urban older adults is insignificant. However, rural older adults had a higher depression level. Rural families have a more intense “duality” contradiction than urban families, and the adaptive choice of living close is more feasible and meaningful. The third explanation is that, according to family support theory, rural daughters provide emotional support and care for their parents instead of sons. In Chinese tradition, sons inherit family businesses and care for their parents (Cong and Silverstein, 2012). Against the backdrop of rural labor migration, women left behind in rural areas take care of their families and actively assume responsibility for caring for their parents (Pianpian et al., 2024). The last explanation is that rural communities exhibit a stronger sense of cohesion compared to urban ones. As a result of the exodus of rural persons, interpersonal relationships in rural communities have become progressively distant, but they may continue to offer substantial support systems for elderly residents.

## 5 Conclusion

We discussed the impact of living alone on depression among older adults based on family culture, which makes some significant contributions to the previous literature. We highlighted that living alone had become the most critical living arrangement for older adults, which improved their depression more positively than expected. Living close to children positively affects rural older adults, while living alone generally appears to be more beneficial for older adults’ mental health than co-residence with children. This fact indicates that China’s family culture has undergone profound changes as individual independence has become part of the family culture and ethics in China. Based on these findings, policymakers should focus on employment. Special emphasis should be placed on incentivizing migrant workers to return to their rural hometowns to start a business or obtain a job.

This study had some limitations. First, other effect factors were not well considered in this research, such as whether social engagement may moderate the effects of living alone on depression among older adults. Moreover, owing to the limitations of the database, some other factors may have remained uncontrolled in this research, such as social support and community environment (Liu and Zhang, 2024; Jia et al., 2023). Finally, the CES-D is a self-report scale that is susceptible to deviations in the evaluation of older adults’ depression levels.

## Data Availability

Publicly available datasets were analyzed in this study. This data can be found here: https://charls.pku.edu.cn/.

## Footnotes

^1^In the traditional intergenerational feedback model, the responsibility for intergenerational care and support has been required, where older adults regard the multigenerational co-residence as a sign of happiness, and the children is required to show respect for their elderly parents, which restrains the impact of intergenerational conflicts from co-residence on depression among older adults.

^2^“Living close” refers to older adults’ living arrangement being in the same city as their children, but not in the same household.

^3^“Living far” refers to older adults’ living arrangement being in different cities than their children.
